# Metagenomics analysis of bacterial community structure from wood- and soil-feeding termites: metabolic pathways and functional structures toward the degradation of lignocellulose and recalcitrant compounds

**DOI:** 10.3389/fmicb.2024.1424982

**Published:** 2024-11-15

**Authors:** Rongrong Xie, Blessing Danso, Jianzhong Sun, Rania Al-Tohamy, Maha A. Khalil, Michael Schagerl, Sameh S. Ali

**Affiliations:** ^1^Biofuels Institute, School of the Environment and Safety Engineering, Jiangsu University, Zhenjiang, China; ^2^Department of Biology, College of Science, Taif University, Taif, Saudi Arabia; ^3^Department of Functional and Evolutionary Ecology, University of Vienna, Vienna, Austria; ^4^Botany Department, Faculty of Science, Tanta University, Tanta, Egypt

**Keywords:** metagenomics, recalcitrant pollutants, termite gut symbionts, bacterial community, operational taxonomic unit, metabolic pathways

## Abstract

Some essential information on gut bacterial profiles and their unique contributions to food digestion in wood-feeding termites (WFT) and soil-feeding termites (SFT) is still inadequate. The feeding type of termites is hypothesized to influence their gut bacterial composition and its functionality in degrading lignocellulose or other organic chemicals. This could potentially provide alternative approaches for the degradation of some recalcitrant environmental chemicals. Therefore, metagenomic analysis can be employed to examine the composition and functional profiles of gut bacterial symbionts in WFT and SFT. Based on the metagenomic analysis of the 16S rRNA gene sequences of gut bacterial symbionts in the WFT, *Microcerotermes* sp., and the SFT, *Pericapritermes nitobei*, the findings revealed a total of 26 major bacterial phyla, with 18 phyla commonly represented in both termites, albeit in varying abundances. Spirochaetes dominated the bacterial symbionts in *Microcerotermes* sp. at 55%, followed by Fibrobacters, while Firmicutes dominated the gut bacteria symbionts in *P. nitobei* at 95%, with Actinobacteria coming in second at 2%. Furthermore, the Shannon and phylogenetic tree diversity indices, as well as the observed operational taxonomic units and Chao 1 richness indices, were all found to be higher in the WFT than in the SFT deduced from the alpha diversity analysis. Based on the principal coordinate analysis, exhibited a significant distance dissimilarity between the gut bacterial symbionts. The results showed that the gut bacterial composition differed significantly between the WFT and SFT. Furthermore, Tax4Fun analysis evaluated bacterial functions, revealing the predominance of carbohydrate metabolism, followed by amino acid metabolism and energy metabolism in both *Microcerotermes* sp. and *P. nitobei* termites. The results implicated that bacterial symbionts inhabiting the guts of both termites were actively involved in the degradation of lignocellulose and other recalcitrant compounds.

## Introduction

Termites are an extraordinarily effective group of lignocellulose-degrading organisms worldwide that contribute to carbon and nitrogen cycling in the ecosystem (Al-Tohamy et al., 2021; Ali et al., 2021; Xie et al., 2024). They transitioned from an omnivore to a wood-feeding lifestyle over 150 million years ago. This was followed by significant modification of their digestive system, including the notable extension of the hindgut and the attainment of cellulolytic flagellates (Brune, 2014). These flagellates are essential for symbiotic digestion in lower termites but disappeared in the most recent lineage, the Termitidae, or “higher” termites, which emerged over 50 million years and possessed an exclusively prokaryotic gut microbiota (Arora et al., 2022). Higher termites are the most varied of all termite families, accounting for nearly 85% of all termite genera. Though higher termites have evolved in various ways, the most significant difference is their diet, which spans beyond “lower termites,” primarily consuming wood (Korb, 2021). Some higher termites ingest sound lignocellulose, such as wood or dried grass, while others consume leaf litter, herbivore dung, humus, and/or soil (Li and Greening, 2022; Xie et al., 2024). The disappearance of symbiotic flagellates in higher termites may be viewed as an evolutionary stride and infer the adoption of a new mechanism for decomposing and obtaining energy from lignocellulose feed (Chouvenc et al., 2021). Although termites secrete endogenous enzymes, their capacity to decompose a lignocellulose diet largely depends on their mutualistic symbiosis with a variety of gut symbionts (Ali et al., 2017; Ali et al., 2019; Ali et al., 2024; Ali et al., 2023). As a result, the study of termite gut symbionts has received much attention in recent years. This aligns with the practical aims of bioprospecting, focusing on industrial lignocellulose conversion and the production of biofuels and other biochemical products (Danso et al., 2022). Despite decades of research, uncovering the gut microbial diversity and its mechanism for lignocellulose degradation has merely been tackled on the surface. There is yet insufficient data to answer the broader ecological questions, including the effect of different feeding regimes on gut microbial community structure and the functional profile of bacterial populations to the hydrolysis of lignocellulose in termites. Noticeably, less molecular data exist for the majority of higher termite species (Marynowska et al., 2020).

More recent research based on culture-independent techniques has provided greater insights into the gut bacterial community structure in wood-feeding termites (WFT) and their potential roles in deconstructing wood (Xie et al., 2024; Nawaz et al., 2023; Santhoshkumar et al., 2024). Nonetheless, the majority of these studies are focused on the *Nasutitermes* species. To fully understand wood degradation in termites, gut microbial communities and their functional profiles in several other species of WFT must be investigated. In addition, soil-feeding termite (SFT) species are the least studied of all termite lineages, though they are the most abundant (Marynowska et al., 2020). Contrary to wood, which basically consists of lignin, cellulose, and hemicellulose, soil contains humus enriched in lignocellulosic polysaccharides as well as amino acids and peptides (Marynowska et al., 2023; Mao et al., 2019). The comprehension of termites to thrive on such a complex diet is still lacking owing to the less studied species of this feeding guild.

Besides, the question of whether termites have distinct microbial symbiotic populations with unique capabilities tailored to various feeding regimes is still completely unanswered. Hence, the purpose of this study was to describe the bacterial community structure inhabiting the gut of different feeding groups of termites and to ascertain if their bacterial symbionts employ different functional structures or mechanisms toward the hydrolysis of lignocellulose and other recalcitrant compounds. The gut bacteria community structure and functional profile of two understudied and most abundant termite species in the southern part of China, the WFT *Microcerotermes* sp., and the SFT *Pericapritermes nitobei*, were investigated in this study employing a culture-independent approach by deploying metagenomics analysis through next generation sequencing of the V3-V4 region of 16S rRNA genes.

## Materials and methods

### Termite collection, identification, and gut extraction

The termite samples used in this study were collected from Xishuangbanna Tropical Botanical Garden, China. Genomic DNA was extracted using a MiniBEST Universal Genomic DNA Extraction Kit (TaKaRa) as described previously (Danso et al., 2022). The cytochrome c oxidase polypeptide II (COII) gene sequencing PCR was performed using universal primers COIIF (5′CAGATAAGTGCATTGGATTT-3′) and COIIR (5′-GTTTAAGAGACCAGTACTTG-3′). The phylogenetic tree was constructed using molecular evolutionary genetics analysis version 7.0 software.

To extract the whole gut, 100 worker-caste of both termite species were placed in a petri dish and sterilized with 70% ethanol, followed by a quick rinse with sterile double-distilled water. The surface-sterilized termites were dissected using sterile instruments under aseptic conditions in laminar airflow. The guts of sterile termites were gently pulled out using sterilized forceps and deposited in a 2 mL Eppendorf tube for metagenomic microbial DNA extraction. Three replicates were prepared for each termite species.

### Amplicon high-throughput sequencing

The extraction of microbial DNA was performed using the HiPure Soil DNA Kits (Magen Guangzhou, China) following the manufacturer’s instructions. The PCR technique was used to amplify the 16S rDNA targeting the V3–V4 region of the ribosomal RNA gene. The amplification process involved subjecting the sample to a temperature of 94°C for 2 min, followed by 30 cycles at 98°C for 10 s, 62°C for 30 s, and 68°C for 30 s. A final extension step was performed at 68°C for 5 min. Specific primers, 341F (5′-CCTACGGGNGGCWGCAG-3′) and 80R (5′-GGACTACHVGGGTATCTAAT-3′), were used for this amplification process according to the standard protocols.

### Bioinformatic and statistical analysis

Raw data containing adapters or low-quality reads were filtered using FASTP to obtain high-quality reads (Chen et al., 2018). Paired-end clean reads were merged as raw tags using FLSAH with a minimum overlap of 10 bp and mismatch error rates of 2% (Magoč and Salzberg, 2011). Noisy sequences of raw tags were filtered to obtain high-quality clean tags by QIIME pipeline under specific filtering conditions (Caporaso et al., 2010). Firstly, the low-quality regions (minimum default quality ≤3; default minimum length ≥ 3) in the raw tags have been identified and split the raw tag at the first low-quality base in the region; secondly, the tag length of a continuous sequence of high-quality bases <75% were removed.

The reference database (version r20110519)^1^ was used to search for clean tags in order to perform reference-based chimaera checking using the UCHIME algorithm (Edgar et al., 2011). All hybrid tags were eliminated, and only efficient tags were retrieved for subsequent study. The efficient labels were grouped into operational taxonomic units (OTUs) with a similarity of at least 97% using the UPARSE pipeline (Edgar, 2013). A representative sequence was chosen from each cluster based on the tag sequence with the highest abundance. OTUs were taxonomically assigned at a confidence threshold of 80% based on the DictDb database (Schloss et al., 2009). Singleton OTUs and sequences identified as chloroplasts or mitochondria were removed from the analysis. OTUs abundance information was normalized with the least sequence number for sample comparison at the same surveying effort. The abundance statistics of each taxonomy were visualized using Krona (Ondov et al., 2011). The stacked bar plot of the community composition was visualized in the R project ggplot2 package (Wickham and Chang, 2008). A heatmap of species abundance in the R project was plotted using the pheatmap package (Kolde and Vilo, 2015). Alpha diversity indices, such as Chao1, Shannon, observed OTUs, and PD-whole tree, were computed using QIIME (Caporaso et al., 2010). The R project ggplot2 package was used to create OTUs rarefaction and rank abundance curves. The alpha index comparison across groups was computed using Welch’s *t*-test in the R project Vegan package (Oksanen, 2010).

To determine the *β* diversity, sequence alignment was performed using Muscle version 3.8.31 (Edgar, 2004), and the phylogenetic tree was constructed using FastTree (Price et al., 2010), then a weighted unifrac distance matrix was generated by the GuniFrac package in the R project (Lozupone and Knight, 2005). The Bray-Curtis distance matrix was calculated in the R project Vegan package (Oksanen, 2010). Multivariate statistical techniques, including principal coordinates analysis (PCoA) of weighted unifrac and bray-Curtis distances, were generated in the R project Vegan package and plotted in the R project ggplot2 package (Wickham and Chang, 2008; Oksanen, 2010). The Kyoto encyclopedia of genes and genomes (KEGG) pathway analysis of the OTUs was inferred using Tax4Fun, which has been shown to provide a good correlation of functional profiles with metagenomic profiles derived from direct sequencing (Aßhauer et al., 2015).

## Results and discussion

### Termite identification and molecular phylogeny

**Figure 1:**
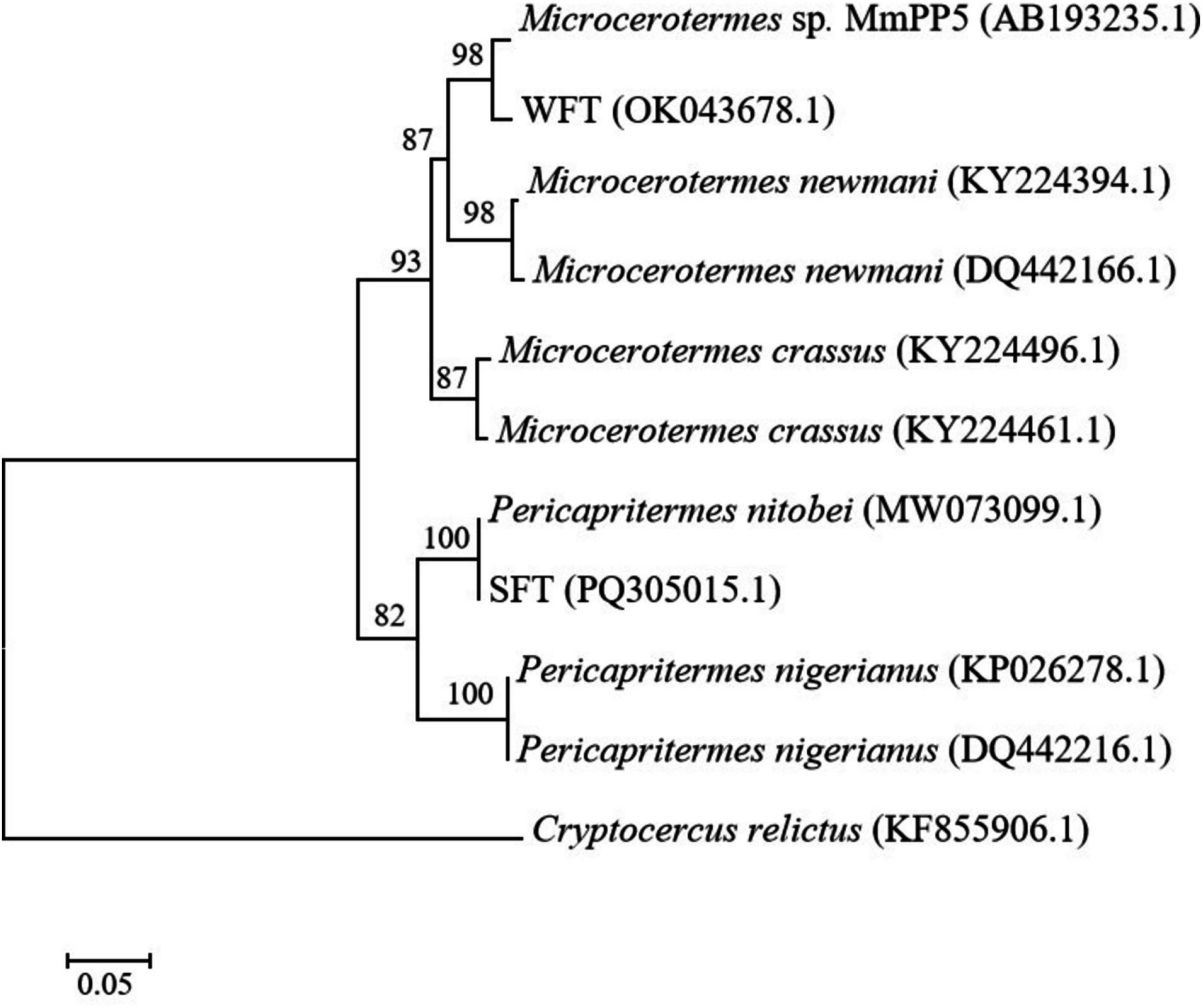
A maximum-likelihood (ML) tree based on the cytochrome oxidase II gene sequences of wood-feeding termites (WFT) and soil-feeding termites (SFT) utilized for this study. *Cryptocercus relictus* was used as an outgroup. The scale bar indicates 0.05 substitutions per nucleotide position. Bootstrap analysis with 1,000 replicates was performed to determine the statistical significance of the branching order. Values at nodes represent bootstrap support values.

A phylogenetic study based on COII gene sequencing was carried out to taxonomically identify the WFT and SFT used in this study. Searching the attained sequences to similar nucleotides in the Basic Local Alignment Search Tool (BLAST) indicated that the termites were affiliated with the most recent family of higher termites, Termitidae. WFT shared the highest similarity with *Microcerotermes* sp. MmPP5 (97.7%), while SFT shared a high similarity of 100% with *Pericapritermes nitobei* (GenBank accession number: MW073099.1). For maximum likelihood phylogeny (Figure 1), the bootstrap values strongly support the branches of the tree, indicating a close relationship between WFT and *Microcerotermes* sp. (with 98% bootstrap support), as well as SFT and *Pericapritermes nitobei* (100% bootstrap support).

### Taxonomic composition of gut bacterial community

**Figure 2:**
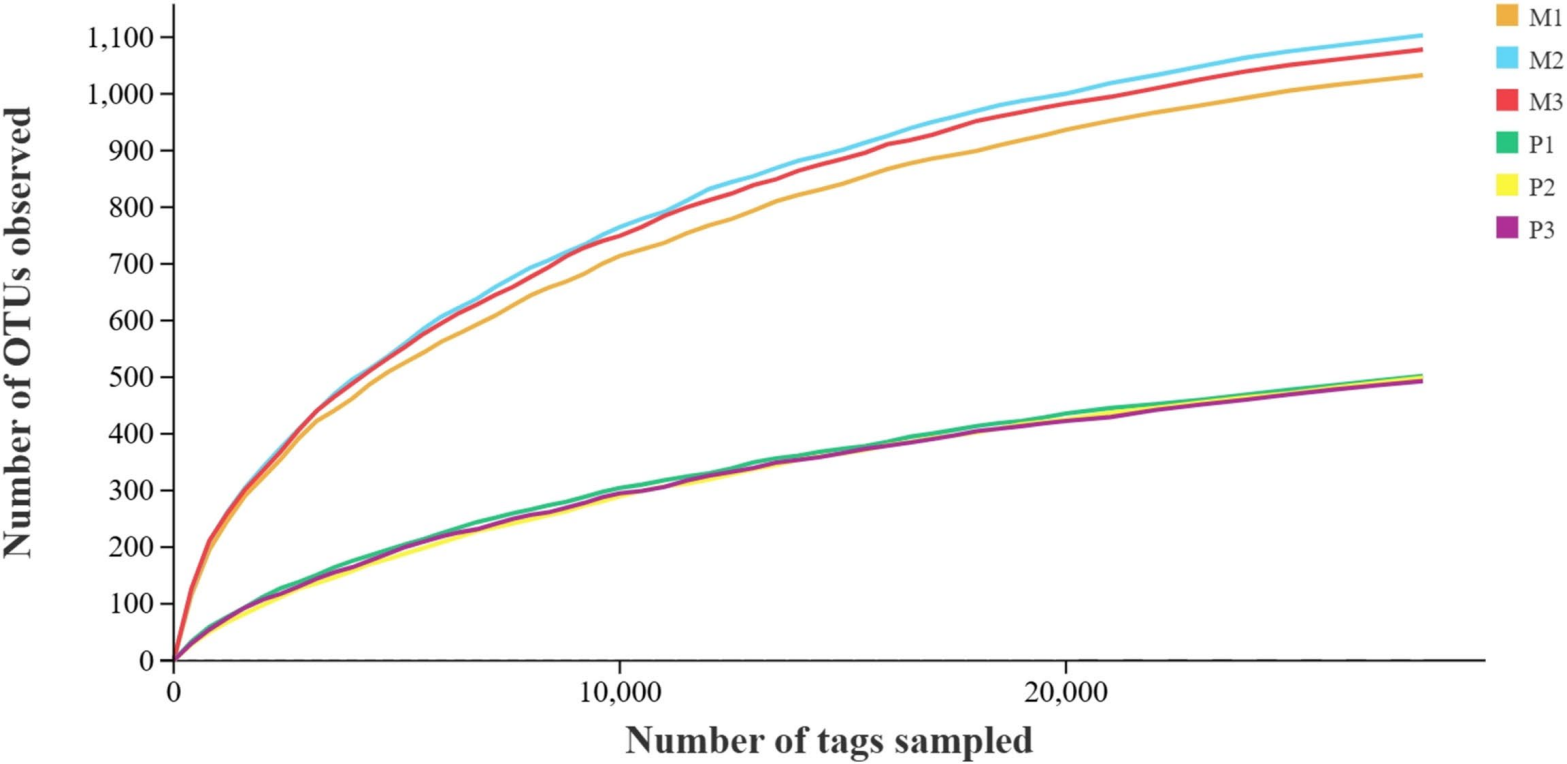
Rarefaction curves of sequence depth of termite gut samples.

A total of 760,285 pair-end reads were obtained from the metagenomic libraries of *Microcerotermes* sp. and *P. nitobei* gut samples by sequencing the V3 and V4 regions of the bacterial communities. The high-quality cleaned reads were further binned and filtered into 547,686 effective tags after removing of chimera, which yielded about 77,438–105,870 effective tags per gut sample. Then, the read number in each sample was normalized to the minimum. A total of 2075 OTUs (at 97% sequence similarity) were identified. Reads were assigned to the phyla and genus taxa using the framework of DictDb. The adequacy of the sampling effort for the bacterial diversity in *Microcerotermes* sp. and *P. nitobei* was assessed by rarefaction analysis. The outcome of this analysis indicated that the rarefaction curves for each termite gut sample reached a plateau, signifying that the sample size was sufficient to accurately reflect the bacterial diversity community (Figure 2).

**Figure 3:**
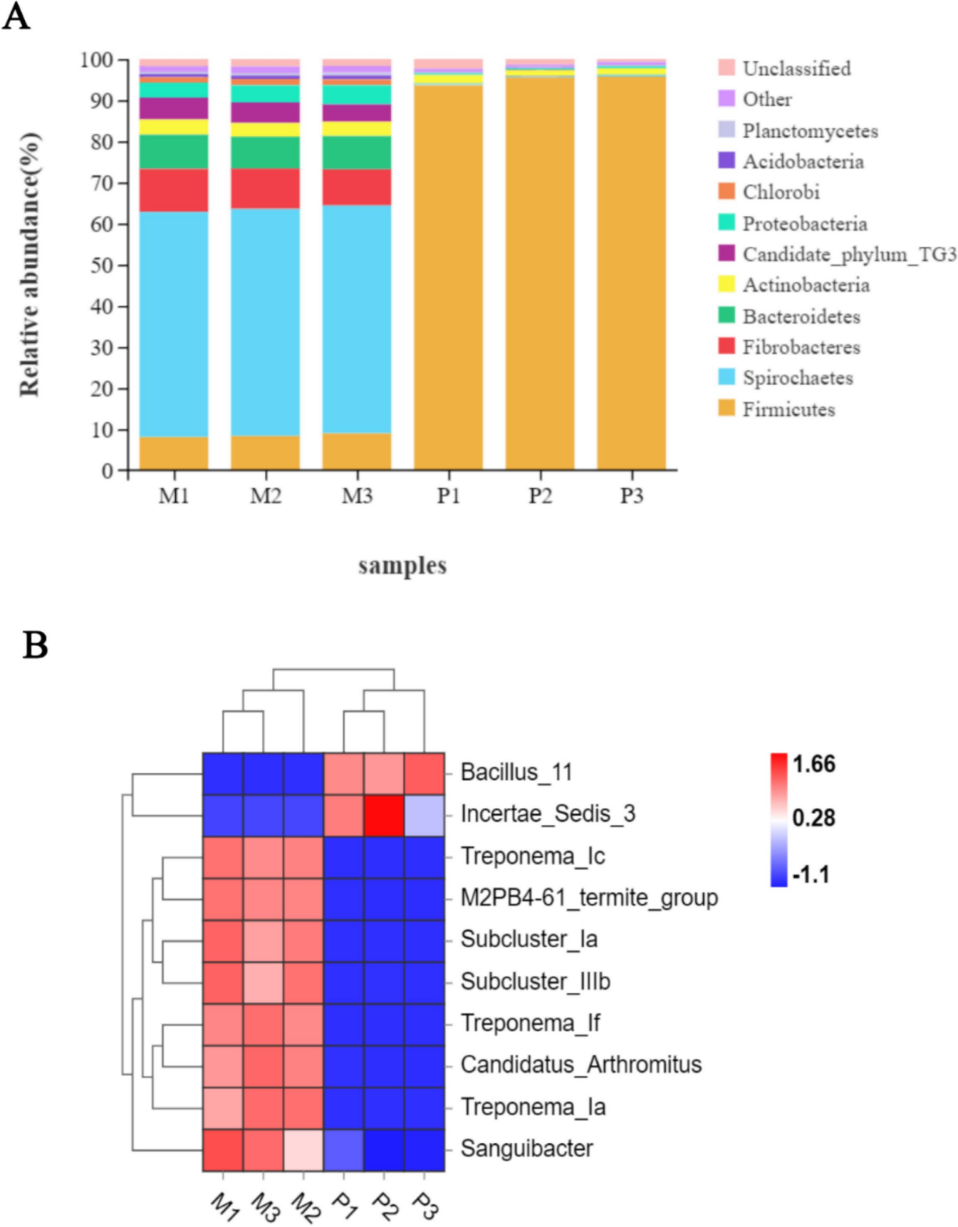
(A) Relative abundance of top 10 bacterial phyla and (B) heat map representation of top 10 genera in the gut of *Microcerotermes* sp. (M1, M2, M3) and *P. nitobei* (P1, P2, and P3).

Following the taxonomic annotation of the resulting bacterial OTUs, 26 bacterial phyla were identified. Among these, 18 phyla were commonly shared between both termites, with relatively higher variations in their relative abundance. The gut system of *Microcerotermes* sp. was dominated by Spirochaetes (Figure 3A), accounting for 55%, followed by Fibrobacteres (10%). Firmicutes and Bacteroidetes were also found in moderate abundance, each representing 8%, followed by Candidate TG3 (4.8%), Proteobacteria (4.1%), and Actinobacteria (3.5%). At the genus distribution, Treponema_1c was the most prevalent and accounted for 28% (Figure 3B). The second most abundant genus was Treponema_1f (11%), followed by Treponema_1a (10.3%), sub-cluster 1a (9.8%), and sub-cluster 1b (4.8%).

In corroboration to the findings in this study, other researchers have reported similar patterns of bacterial community structure in the gut of several WFT, including *Nasutitermes* species, *Globitermes brachycerastes*, *Microcerotermes strunckii*, *Mironasutitermes shangchengensis*, and *Microcerotermes* species (Warnecke et al., 2007; Liu et al., 2019; Makonde et al., 2015). Spirochetes are represented across several termites and have been observed to perform essential metabolic activities essential for termites (Manjula et al., 2016). In addition, Spirochaetes were found in the guts of SFT *Nasutitermes corniger* and WFT *Nasutitermes takasagoensis* as part of the wood fiber-associated cellulolytic bacterial community (Mikaelyan et al., 2014). Consequently, the prevalence of Spirochaetes populated by clusters of Treponema in the WFT, *Microcerotermes* sp., may result from the carbohydrate-rich diet. Besides, recent metagenomic and metatranscriptomics analysis of gut bacterial symbionts has identified Spirochaetes of the Treponema genus, Fibrobacters, and Firmicutes to encode several putative CAZymes (including cellulases and hemicellulases) for lignocellulose degradation (Marynowska et al., 2020; Liu et al., 2019). On the other hand, the phyla Firmicutes (95%) (Figure 3A) populated the gut bacterial community in the SFT, followed by Actinobacteria (2%). Proteobacteria and Candidate TG 3 accounted for 0.43% each, followed by Planctomycetes (0.19%), Chloroflex (0.13%), and Acidobacteria (0.11%). The genus representatives in *P*. *nitobei* were dominated by Bacillus_11 (73%) (Figure 3B). Incertae _sedis_3 (15%) accounted for the second most populous genus, followed by Sporosarcina_5 (1.23%) and Gut_cluster_13 (0.58%).

The abundance of Firmicutes in *P. nitobei* is consistent with preliminary literature on SFT such as *Cubitermes niokoloensis*, *Labiotermes labralis*, and *Syntermes* sp., *Cubitermes ugandensis*, and *Termes riograndensis* (Marynowska et al., 2020; Liu et al., 2019; Fall et al., 2007). The presence of the Firmicutes phyla, namely the *Bacillus* species, in the digestive system of termites has been demonstrated to play a role in breaking down cellulose, obtaining nitrogen, and producing acetate (Manjula et al., 2016). This is due to their mutually beneficial interaction with the termites as endosymbionts. It has also been observed that several *Bacillus* species identified from insects and other environments could efficiently degrade lignin and other recalcitrant aromatic compounds (Mei et al., 2020); thus, they are considered to contribute to the degradation of lignin and recalcitrant compounds in termites. Additionally, metagenomic sequencing revealed that Firmicutes partake in the secretion of cellulases, hemicellulases, and peptidases in termites (Marynowska et al., 2020). The abundance of Firmicutes phyla in SFT may be influenced by the rich content of organic matter present in the soil diet as well as the high alkalinity of the gut system. Likewise, Mikaelyan et al. (2017) identified Firmicutes as the most prevalent phyla in the most alkaline compartment of several termites, such as *Termes hospes, Cornitermes* sp., *Microcerotermes parvus*, and *Neocapritermes taracua*. Several other phyla, including Proteobacteria, Actinobacteria, Planctomycetes, and Chlorobi, are also implicated in processing lignocellulose components in termites (Diouf et al., 2015). Moreover, Actinobacteria are noted to produce secondary metabolites that offer protection to the termites (Kurtböke et al., 2015).

A potential difference in the composition of the bacterial community related to diet has also been observed in WFT, including *Microcerotermes strunckii* and *Nasutitermes corniger,* where Spirochaetes dominated and SFT like *Termes riograndensis* populated by Firmicutes (Vikram et al., 2021). Similarly, He et al. (2013) identified Spirochaetes and Fibrobacters to dominate in the hindgut of WFT, *Nasutitermes corniger,* whereas Firmicutes were prevalent in the dung-feeding termite, *Amitermes wheeleri*. Although these studies depict diet as a primary determinant of gut composition in termites, certain studies have also related the difference in gut bacterial composition to the co-evolution of the termite and the host environment (Abdul Rahman et al., 2015). The variations in bacterial community structure observed in the present study could be associated with the shift in diet during the evolutionary process, such that dietary influences on gut microbiota were prominent by colonizing specific bacterial lineages to adjust to the new diet. Another co-factor is the difference in physiological conditions prevailing in their gut system. Previous studies have established that the gut system of SFT is morphologically elongated and possesses an elevated pH than wood-feeders; thus, it may be dominated by certain bacterial phylotypes like Firmicutes that can survive in the gut environment (Mikaelyan et al., 2017). The findings from this study corroborate with existing literature and implicate that microbial community structures differ in different feeding groups of termites.

### Diversity of gut bacterial composition between *Microcerotermes* sp. and *P. nitobei*

**Figure 4:**
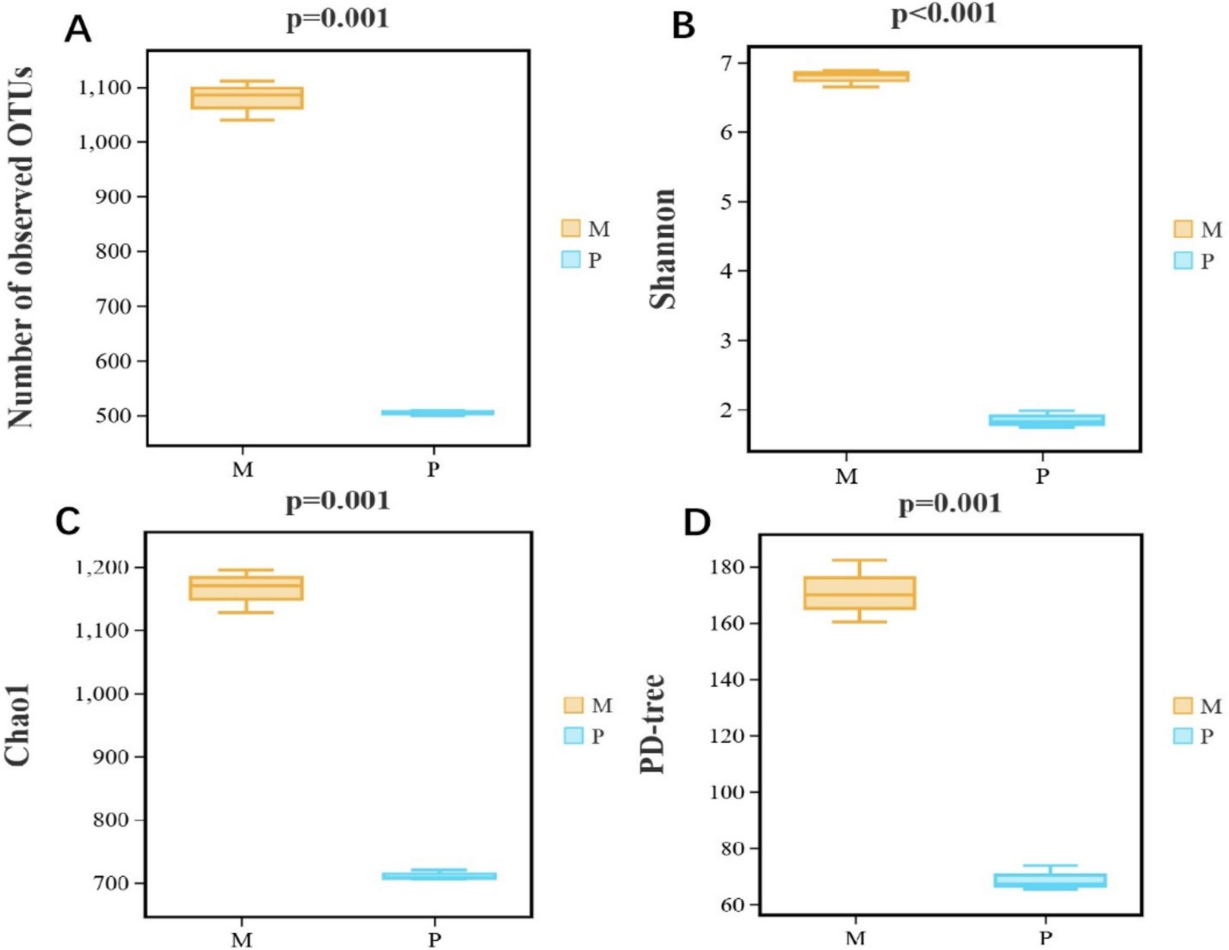
Bacterial *α*-diversity measures. (A) Number of observed OTUs, (B) Shannon, (C) Chao1, and (D) PD-tree of *Microcerotermes* sp. (M) and *P. nitobei* (P) termite species.

The bacterial diversity and richness were analyzed employing alpha diversity based on observed OTUs, Shannon, Chao1, and PD tree indices. The observed OTUs (*p* = 0.001), Shannon 1 (*p* ≤ 0.001), Chao1 (*p* = 0.001), and PD tree (*p* = 0.001) indices of *Microcerotermes* sp. were all significantly higher than those of *P. nitobei* (Figure 4), implicating that WFT, *Microcerotermes* sp., had the highest community diversity and community richness in contrast to SFT, *P. nitobei*. For *P. nitobei,* two OTUs (OTU000001 and OTU000002) assigned to Bacillaceae and Planococcaceae were the most abundant members, accounting for more than 85% of all sequence reads in each sample, while the same was represented by 133 OTUs for *Microcerotermes* sp. The lower bacterial diversity and richness detected in the SFT may result from the preservation of a more specialized microbiota required for the effective digestion of soil organic matter and other aromatic subunits for the survival of the host, as well as the high alkalinity of the gut system. However, results from this study contradict previous studies, which reported higher microbial diversity and richness in the SFT contrary to the WFT and related it to the diverse range of carbon and nitrogen sources available in soil feeders’ diets compared to the limited carbohydrate-rich diet for the wood feeders (Vikram et al., 2021). Nonetheless, the highest diversity observed in the WFT compared to the SFT in this study may be influenced by host phylogeny and host habitat.

**Figure 5:**
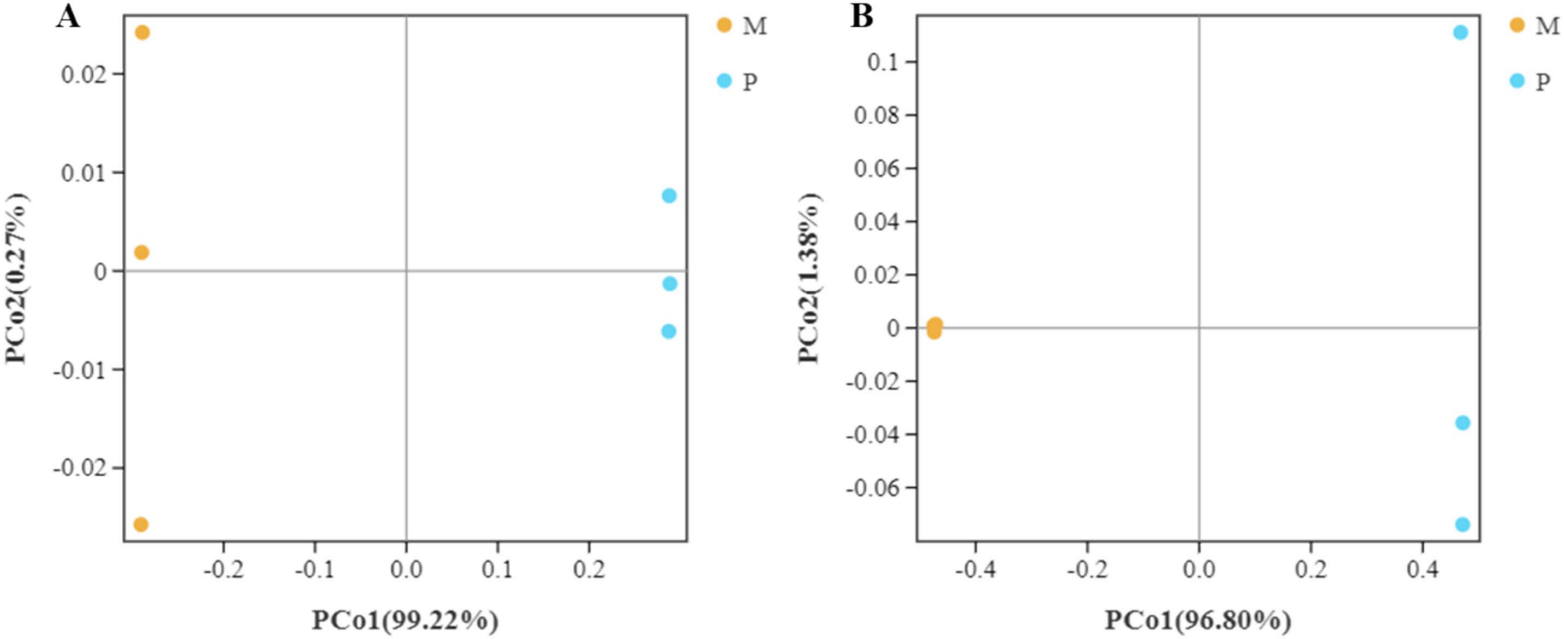
Bacterial *β* diversity. Principal coordinate analysis (PCoA) of pairwise distances among bacterial communities based on (A) weighted Unifrac and (B) Bray-Curtis distances. The yellow color represents replicate samples from the wood feeding termite, *Microcerotermes* sp. (M), and the color blue reflects repeated samples from soil feeding termite, *P. nitobei* (P).

The distance between the bacterial community structure of the WFT and SFT species was assessed using the beta diversity analysis based on the weighted UniFrac and Bray-Curtis distances. The UniFrac distances of the triplicate samples of gut bacterial composition in SFT were found to be similar but were significantly distance from the bacterial community structure in the WFT (Figure 5A). Furthermore, the Bray-Curtis distance analysis revealed that the triplicate gut samples from WFT shared high similarity in bacterial composition structure and clustered significantly away from the community in WFT (Figure 5B). A similar trend was also shown by the Bray-Curtis (R2 = 0.96, *p* < 0.01) and weighted UniFrac distance (R2 = 0.98, *p* < 0.01) based on the Adonis (PERMANOVA) analysis. Both analyses demonstrate the significant variation of *β* diversity between the WFT and SFT. Significant differences between the gut bacterial community structure in *Microcerotermes* sp. and *Pericapritermes nitobei* have been exhibited. This may be attributed to the evolutionary transition, which caused a change in the composition of gut symbiont to assist the host termite in adapting to their new habitat and feed type (Rossmassler et al., 2024).

### Predicted functional profile of the bacterial communities in the gut of *Microcerotermes* sp. and *P. nitobei*

To predict a potential functional composition of the bacterial symbionts in termite guts, a Tax4Fun program was applied to each bacterial sample. The Tax4Fun analysis has been shown to provide a good correlation of predicted functional profiles with metagenomic profiles derived from direct sequencing (Aßhauer et al., 2015). So far, functional profiles using Tax4Fun have been predicted from 16S rRNA gene datasets derived from various environments (Li et al., 2024; Pratama et al., 2019).

**Figure 6:**
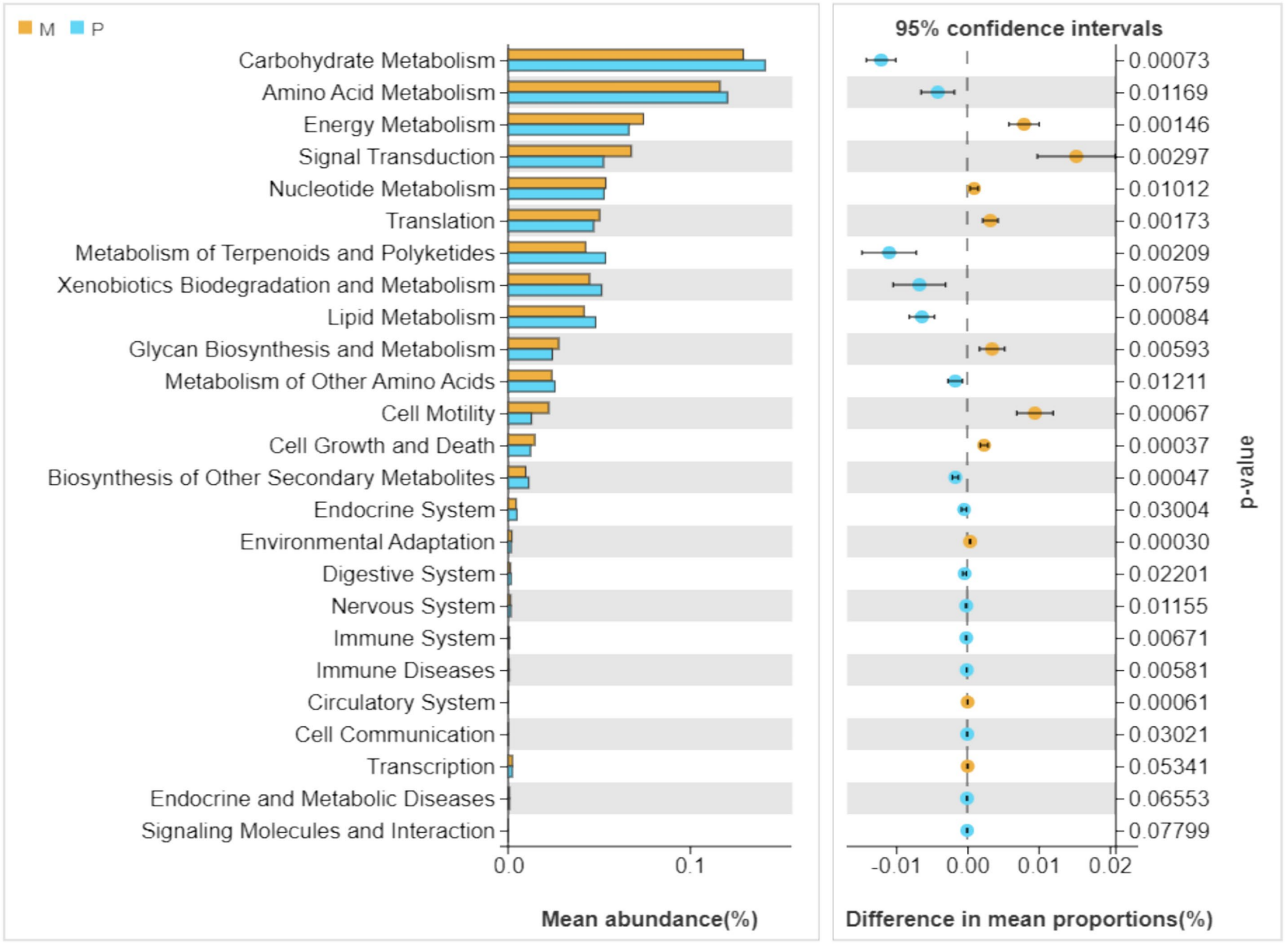
Relative abundances (top 20) of the putative metabolic pathways in the gut bacteria of *Microcerotermes* sp. (M) and *Pericapritermes nitobei* (P). The Tax4Fun program was used to predict the functional capabilities of the microbial communities based on 16S rRNA gene amplicon sequencing data. The significantly varied metabolic pathways between wood feeding and soil-feeding termites are indicated by *p* ≤ 0.05. Values are presented as the mean (*n* = 3).

As depicted in Figure 6, the dominant metabolic pathways of gut bacterial symbionts in *Microcerotermes* sp. and *P. nitobei* were indeed characterized as carbohydrate, amino acid, and energy metabolisms, which suggested that the gut bacterial symbionts might play a vital role in host nutrition and energy supply (Shapira, 2016). A PCoA analysis was performed in terms of the obtained KEGG functional profiles, where the samples from *Microcerotermes* sp. formed an obvious and independent cluster separated from that of *P. nitobei* (Supplementary Figure S1). The taxonomic differentiation and the accordingly associated functional differentiation of the symbiotic bacterial community of WFT and SFT presented here suggest a possible type of ecological adaptation mechanism from termites. Statistical analysis employing the Welch’s *t*-test was carried out to determine the significant (*p* ≤ 0.05) abundance of metabolic pathways between *Microcerotermes* sp. gut bacterial symbionts and that of *Pericapritermes nitobei*. Of all the metabolic functions detected, those associated with carbohydrate metabolism were the most abundant in both *Microcerotermes* sp. and *Pericapritermes nitobei* gut bacteria symbionts (Figure 6), reflecting their functional importance for the degradation of their lignocellulose diet. Further statistical analysis indicated that the carbohydrate metabolism pathway was significantly (*p* ≤ 0.05) enriched in the SFT, *P. nitobei* gut bacteria symbionts, compared to the WFT, *Microcerotermes* sp. (Supplementary Figure S2). This outcome is contrary to previous findings, which reported gut bacterial symbionts of several WFT to be enriched in carbohydrate metabolism and encode abundant carbohydrate-active enzymes (CAZymes) than SFT owing to the rich carbohydrate diet of WFT (Arora et al., 2022). The abundance of carbohydrate metabolism pathway from the gut symbionts of *P. nitobei,* contrary to *Microcerotermes* sp., is quite interesting and may result from the abundance of non-cellulosic polysaccharides such as chitin in the soil diet. This is further supported by the observation of Marynowska et al. (2020), who reported the high diversity of carbohydrate-active enzymes in the SFT, *Labiotermes labralis*. In this study, the more abundant carbohydrate metabolic pathway in *P. nitobei* may be related to the dominant *Bacillus* (average abundant 73%) in this termite species, which has been demonstrated to play an important role in breaking down carbohydrate (Zhao et al., 2024; Malik and Javed, 2021; Dar et al., 2021; Bisht et al., 2020). However, the significant (*p* ≤ 0.05) abundance of glycan biosynthesis and metabolism pathways detected in WFT is consistent with previous findings. Generally, wood has a higher content of stored polysaccharides than soil. Therefore, the enrichment of glycan biosynthesis and metabolism pathway from the gut bacteria community in *Microcerotermes* sp. than *P. nitobei* is consistent with their diet. In addition, the enrichment of cell motility in *Microcerotermes* sp. may be related to the dominant of *Treponema* Ic and If, which can move to wood fibers for cellulose hydrolysis (Mikaelyan et al., 2014).

In contrast to wood, soil contains a variety of recalcitrant and nitrogenous compounds originating from the humic components (peptides, aromatic subunits, amino acids, microbial excretions, and plant debris). Thus, several KEGG pathways, including amino acid metabolism, metabolism of terpenoids and polyketides, xenobiotic degradation and metabolism, lipid metabolism, and metabolism of other secondary metabolites, were significantly (*p* ≤ 0.05) enriched in the *P. nitobei* gut bacterial community. Similarly, the SFT *Cubitermes orthognathus* has been observed to metabolize peptidoglycan and other microbial excretion components at a higher rate than cellulose (Ji and Brune, 2001). In addition, recent findings by Rossmassler et al. identified gut symbionts of the P3 compartment of *Cubitermes ugandensis*, SFT to encode more peptidase genes than those in *Nasutitermes corniger* and *Microcerotermes parvus* WFT (Rossmassler et al., 2024). Though bacteria composition and community structure significantly differed between the WFT and SFT in this study, the symbiotic gut bacteria communities demonstrated a similar functional structure toward the degradation of lignocellulose and recalcitrant compounds.

## Conclusion

This study employed metagenomic analysis to investigate the bacterial communities and functional profiles of understudied termite species, *Microcerotermes* sp. and *P. nitobei*. In the gut of *Microcerotermes* sp., the bacteria phylum Spirochetes (mainly the genus Treponema clusters) were the most common. In the gut of *P. nitobei*, on the other hand, Firmicutes (mainly the *Bacillus* genus) were the most common. Further analysis revealed that the diversity of bacteria was higher in the WFT than in the SFT. Furthermore, the *β*-diversity analysis revealed significant differences between the gut bacterial community structures of *Microcerotermes* sp. and *P. nitobei*. Furthermore, the functional metabolic pathways of gut bacteria from both termites were assessed. The bacterial communities were enriched in carbohydrate metabolism independent of feeding behavior. Ultimately, the outcome of this study depicts that the individual termite gut system contains unique bacterial flora yet exhibits a functional congruence toward lignocellulose degradation and other recalcitrant compounds. Although the metagenomic study provided an overview of the bacterial community structure and potential metabolic pathways associated with lignocellulosic biomass degradation, such an approach does not allow for estimating microbial physiology or ecology. Furthermore, cultivable microbes are suitable for studying symbiotic relationships within the host and exploring their potential applications. Thus, enrichment techniques are under investigation to ascertain the composition of culturable lignocellulolytic gut bacteria symbionts in *Microcerotermes* sp. and *P*. *nitobei*.

## Data Availability

The datasets presented in this study can be found in online repositories. The names of the repository/repositories and accession number(s) can be found in the article/Supplementary material.

## Footnotes

^1^ http://drive5.com/uchime/uchime_dowload.html
