# Supplementary material for: Metagenomics analysis of bacterial community structure from wood- and soil-feeding termites: metabolic pathways and functional structures toward the degradation of lignocellulose and recalcitrant compounds
Source: Front Microbiol. 2024 Nov 15;15:1424982. doi: 10.3389/fmicb.2024.1424982 (PMC11604708; doi:10.3389/fmicb.2024.1424982)
Supplement: Supplementary file 1 [file Supplementary_file_1.docx]

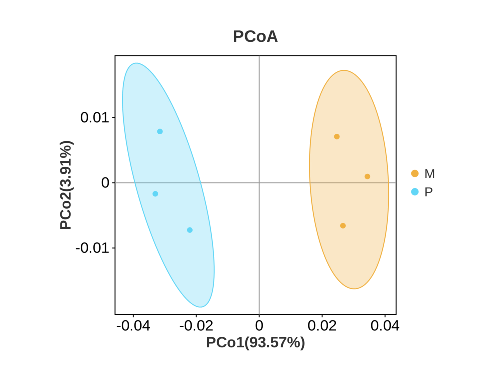


**Fig. S1.** Principal coordinates analysis (PCoA) on bacterial KEGG functional profiles of the host termite *Microcerotermes* sp. (M) and *Pericapritermes nitobei* (P) in terms of Bray-Curtis.


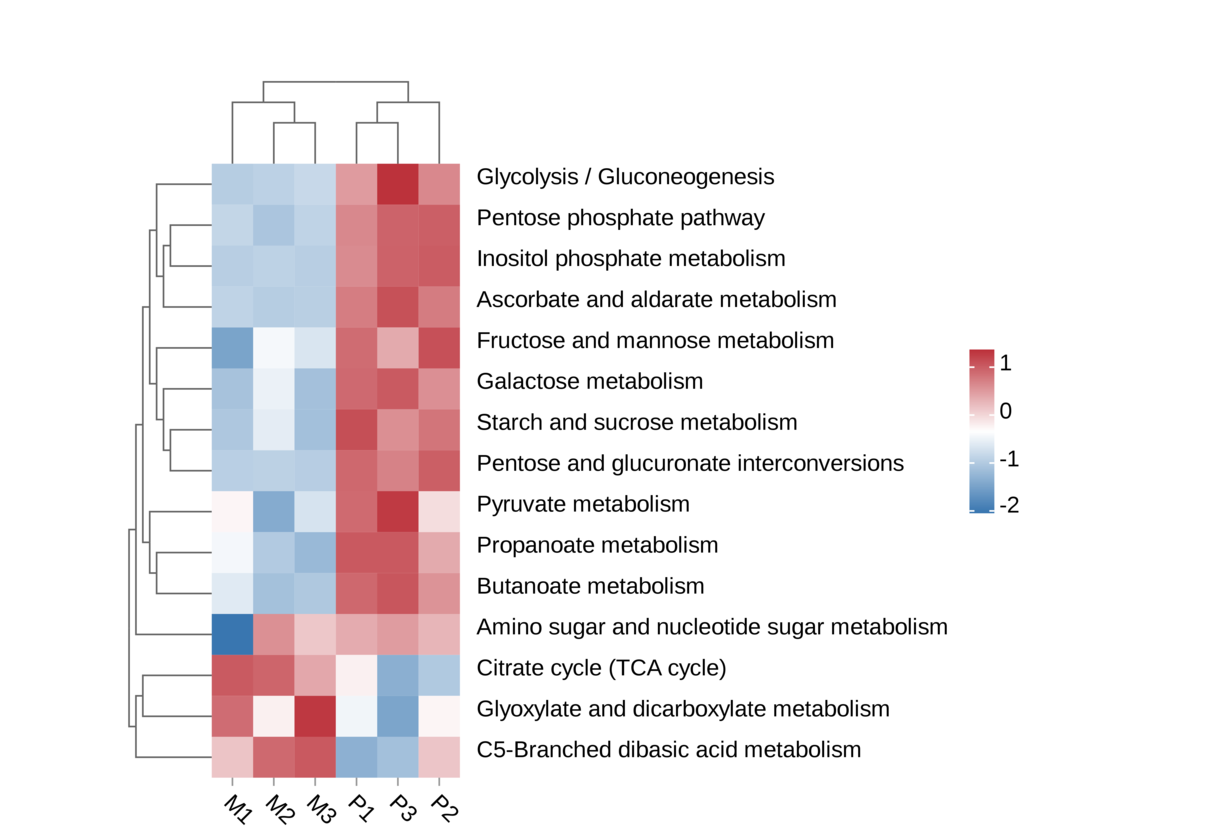


**Fig. S2.** Heatmap of the relative abundance of modules related to carbohydrate metabolism identified in prokaryotic microbiomes of *microcerotermes* sp. (M) and *Pericapritermes nitobei* (P).
